# Dectin-1 signaling coordinates innate and adaptive immunity for potent host defense against viral infection

**DOI:** 10.3389/fimmu.2023.1194502

**Published:** 2023-06-02

**Authors:** Hyeong Won Kim, Mi-Kyeong Ko, So Hui Park, Seong Yun Hwang, Dong Hyeon Kim, Sun Young Park, Young-Joon Ko, Su-Mi Kim, Jong-Hyeon Park, Min Ja Lee

**Affiliations:** Center for Foot-and-Mouth Disease Vaccine Research, Animal and Plant Quarantine Agency, Gimcheon-si, Gyeongsangbuk-do, Republic of Korea

**Keywords:** foot-and-mouth disease, Dectin-1 signaling, β-D-glucan, adjuvant, innate and adaptive immunity, host defense, viral infection

## Abstract

**Background:**

Most commercial foot-and-mouth disease (FMD) vaccines have various disadvantages, such as low antibody titers, short-lived effects, compromised host defense, and questionable safety.

**Objectives:**

To address these shortcomings, we present a novel FMD vaccine containing Dectin-1 agonist, β-D-glucan, as an immunomodulatory adjuvant. The proposed vaccine was developed to effectively coordinate innate and adaptive immunity for potent host defense against viral infection.

**Methods:**

We demonstrated β-D-glucan mediated innate and adaptive immune responses in mice and pigs *in vitro* and *in vivo*. The expressions of pattern recognition receptors, cytokines, transcription factors, and co-stimulatory molecules were promoted *via* FMD vaccine containing β-D-glucan.

**Results:**

β-D-glucan elicited a robust cellular immune response and early, mid-, and long-term immunity. Moreover, it exhibited potent host defense by modulating host’s innate and adaptive immunity.

**Conclusion:**

Our study provides a promising approach to overcoming the limitations of conventional FMD vaccines. Based on the proposed vaccine’s safety and efficacy, it represents a breakthrough among next-generation FMD vaccines.

## 1 Introduction

Foot-and-mouth disease (FMD) primarily affects animals with cloven hooves, such as cattle, sheep, and pigs. FMD causes considerable economic losses in the livestock industry because of its widespread transmission and large-scale effect on productivity. The FMD virus (FMDV) can be transmitted through various routes, including *via* contact with animal handlers, infected animals, inanimate objects, such as feed or vehicles, and even through aerosolized transmission (1).

The currently available FMD vaccines induce a humoral immune response; however, they have several limitations. First, conventional vaccines take 14–21 d to generate a sufficient adaptive immune response and do not defend the host against viral infection in the early stage of vaccination. Second, because current FMD vaccines have a short duration of effect, routine vaccination is required. Third, side effects, such as fibrosis and granuloma, frequently occur at the site of intramuscular (IM) injection in pigs. An ideal vaccine should induce early, mid-term, and long-term immunity by simultaneously inducing cellular and humoral immune responses, elicit potent host defenses, reduce or eliminate side effects, and be optimized for various livestock species (2).

FMD vaccine uses inactivated whole virus as an antigen, and an adjuvant is used to increase the efficacy of the vaccine. Unlike live attenuated virus vaccines, eliciting a strong and long-lasting immune response with an inactivated viral antigen often requires the addition of a potent and safe adjuvant (3). Many types of adjuvants are used in commercial animal vaccines, but aluminum hydroxide-based and mineral oil-based adjuvants with or without saponins are most commonly used for inactivated FMD vaccines.

Aluminum hydroxide (Gel), Quil-A (saponin), and ISA 206 (W/O/W emulsion) have been used as FMD vaccine adjuvants in our previous studies (2, 4).

During the manufacturing process of FMD vaccine, the inactivated antigen is adsorbed on aluminum hydroxide. After adsorption, the antigen is aggregated on the surface and inside of the aluminum hydroxide-based adjuvant particle to help maintain the physicochemical properties of the antigen. Adjuvant particles elicit an immune response by presenting the relocated antigen to immune cells and facilitating interactions between antigens and immune cells over a long-lasting period, which is a phenomenon called “repository effect” (also called “depot effect”) (5, 6). Uptake of antigens by antigen-presenting cells (APCs) is pivotal for the induction of immune responses. After the vaccine is injected into the body, the antigen adsorbed on aluminum interacts with APC to cause a primary immune response. Along with the decomposition of aluminum hydroxide, the antigen inside the aluminum hydroxide-based adjuvant is gradually released, delaying the consumption of the antigen and prolonging the stimulation period of the immune system (7). The longer the interval between interactions between APCs and antigens, the better the immune response. The “repository effect” has long been accepted as one of the mechanisms behind the ability of aluminum hydroxide-based adjuvants to stimulate an immune response (8).

Aluminum hydroxide-based adjuvants have been in use for nearly 100 years, and more than one billion safe injections have been given to adults and children for aluminum hydroxide adsorbed vaccines, such as DTP and hepatitis B vaccines. However, aluminum hydroxide-based adjuvants have been found to have some drawbacks. For example, vaccination with aluminum hydroxide-based adjuvant vaccines can cause local adverse reactions such as erythema, subcutaneous nodules, contact hypersensitivity, and granulomas (9).

Another downside to aluminum hydroxide-based supplements is that they cannot be stored frozen. Antigens in vaccines containing aluminum hydroxide-based adjuvants are adsorbed and supported by grid structures of aluminum salts that are prone to destruction upon freezing. Therefore, aluminum hydroxide-based adjuvant vaccines cannot be stored below 0°C (10, 11). In addition, vaccines containing aluminum hydroxide and saponin as adjuvants have several deficiencies, such as requiring relatively frequent revaccination at 6 or even 4-month intervals due to the short lifespan of antibodies (antibody maintenance period).

In contrast, FMD vaccines containing oil-based adjuvants appear to provide more effective protection against viral infection, with several advantages, such as the induction of high antibody titers and long-lasting antibody responses (12, 13). In addition, unlike aluminum hydroxide-based adjuvant vaccines, oil-based adjuvant vaccines can overcome interference by maternally-derived antibodies in neonates (young individuals), enabling early application (14).

β-D-glucan occurs naturally in the cell walls of bacteria and fungi and elicits different immune responses depending on its molecular weight, shape, and structure (15). For example, intestinal cells induce the migration of β-(1,3)-glucan *via* the intestinal cell wall to the lymph, and the lymph stimulates macrophages (MΦs) to activate the host’s immune (16).

Dendritic cell-associated C-type lectin (Dectin)-1 is a pattern recognition receptor (PRR) that recognizes groups of β-glucans, including β-D-glucans. Dectin-1 is a protein encoded by the C-type lectin domain-containing (CLEC) 7A gene in humans, a membrane receptor with a C-type lectin-like domain fold, and a partial immune receptor tyrosine-based activation motif (17). It acts as a PRR for various β-glucans, including β-D-glucan, and plays a crucial role in the innate immune response, representing the first line of host defense. Dectin-1 is expressed in MΦs, eosinophils, dendritic cells (DCs) and B cells (18, 19). The caspase recruitment domain (CARD)-containing coiled-coin (CC) protein/B cell lymphoma (BCL)-10/mucosa-associated lymphoid tissue lymphoma translocation protein (MALT)-1 (CBM) signaling complex induced by the activation of Dectin-1 promotes tumor necrosis factor receptor-associated factor (TRAF) 6 and nuclear factor kappa B (NF-κB) activation (20, 21). Consequently, NF-κB induces the production of numerous inflammatory cytokines, such as tumor necrosis factor (TNF), interleukin (IL)-23, IL-6, and IL-2, as well as chemokines, such as monocyte chemotactic protein (MCP)-1, regulated upon activation normal T cell expressed and presumably secreted (RANTES), macrophage inflammatory protein (MIP)-2, C-X-C motif ligand (CXCL) 1, and CXCL10 (22). Dectin-1 can recognize the endogenous ligands of and bind to both CD4^+^ and CD8^+^ T cells to induce cell activation and proliferation (18, 23).

In this study, we designed a novel FMD vaccine incorporating β-D-glucan as an adjuvant to induce innate and adaptive (cellular and humoral) immune responses as well as systemic and mucosal immunity. We evaluated the efficacy and safety of the β-D-glucan-containing FMD vaccine *in vitro* (in murine resident peritoneal cells [RPCs] and porcine peripheral blood mononuclear cells [PBMCs]) and *in vivo* (mice and pigs), and elucidated the underlying mechanism.

## 2 Materials and methods

### 2.1 Antigen purification from FMDV type O (O PA2) and type A (A YC)

Given that FMDV types O and A are problematic variants in East Asia, including Korea, we selected the antigens derived from O/PKA/44/2008 (O PA2) and A/SKR/YC/2017 (A YC), which show excellent yield and antigenicity, in our analyses (**Supplementary Figures 1, 2**).

To produce purified antigen (inactivated FMDV), BHK-21 cells infected with FMDV O PA2 and A YC were used as designed by Lee et al. (4). For FMDV infection, the culture medium of BHK-21 cells was replaced with a serum-free culture medium, and the virus was inoculated and incubated at 37°C with 5% CO_2_ for 1 h. DMEM (HyClone, Logan, UT, USA) was used as the culture medium for BHK-21 cells. The viruses were harvested after complete cytopathic effect (CPE) was observed in infected BHK-21 cells, followed by centrifugation at 12000 rpm for 20 min to remove cell debris and obtain viral supernatants. Thereafter, the viruses were inactivated using 0.003 mM of binary ethylenimine (BEI, Sigma-Aldrich, St. Louis, MO, USA) for 24 h, and concentrated using 7.5% polyethylene glycol (PEG) 6000 and 2.3% NaCl, followed by stirring for 16 h at 4°C (24, 25).

The concentrated pellet was resuspended in Tris-NaCl buffer and purified by a 15–45% sucrose gradient at 30,000 rpm for 4 h at 4°C using an SW41Ti rotor (Beckman Coulter, Brea, CA, USA). Afterward, the 146S antigen quantity was measured at 259 nm using a spectrophotometer (BioSign FMDV Ag; Princeton BioMeditech, Princeton, NJ, USA). The inactivated virus was passaged at least three times in ZZ-R 127 and BHK-21 cells to confirm the absence of live virus before use in experiments (4, 25).

### 2.2 Confirmation of structural and non-structural proteins using purified antigens and examination of 146S particles using transmission electron microscopy (TEM)

The expression of structural proteins (SPs) of the purified antigen was demonstrated in cells infected with FMDV O PA2 and A YC antigen using rapid antigen kits (PBM kit, Princeton BioMeditech); SPs were confirmed by band formation and non-structural proteins (NSPs) by no band formation (**Supplementary Figure 1A, B**). Viral particles (146S) were characterized using TEM imaging (**Supplementary Figures 2A, B**) (4).

### 2.3 Mice and pigs

Animal experiments were performed using C57BL/6 mice (female, 6–7 weeks-old) purchased from KOSA BIO (Gyeonggi-do, Korea) and pigs (8–9 weeks-old) as previously described (4, 26). All the mice and pigs were housed in animal biosafety level 3 (ABSL3) at the Animal and Plant Quarantine Agency (APQA, Gyeongsangbuk-do, Korea), and acclimatized for one week prior to the experiment. The animal study was conducted in accordance with institutional guidelines and was approved by the APQA Ethics Committee (accreditation number: IACUC-2022-659).

### 2.4 RPCs isolation and culture

Murine RPCs were isolated from the peritoneal lavage fluid of naive mice as previously described (2, 4). Naive mice (n = 40) were anesthetized using CO_2_ and sacrificed by cervical dislocation. The abdominal cavity was flushed with 5 mL Ca^2+^/Mg^2+^-free DPBS (Gibco, Waltham, MA, USA). The collected peritoneal lavage fluid was centrifuged at 400 g for 10 min at 4°C, and the pelleted RPCs were resuspended in complete RPMI-1640 (Gibco) medium [RPMI-1640 medium containing 0.05 mM 2-β-mercaptoethanol (Sigma-Aldrich), 3 mM L-glutamine (Sigma-Aldrich), 10 mM HEPES (Sigma-Aldrich), 100 U/mL penicillin/streptomycin (Sigma-Aldrich), and 10% fetal calf serum (HyClone)] and incubated at 37°C with 5% CO_2_ (4). All cells were freshly isolated prior to use.

### 2.5 PBMCs isolation and culture

PBMCs were individually isolated from the whole blood samples (20 mL/donor) collected from FMD antibody-negative pigs (8–9 weeks-old, n = 3/group) in heparin tubes (Becton, Dickinson and Company, Franklin Lakes, NJ, USA), using Histopaque-1077 (Sigma-Adrich) as previously described (4, 25). The remaining red blood cells were then removed by lysis using ACK lysing buffer (Gibco). Thereafter, PBMCs were resuspended in Ca^2+^/Mg^2+^ free DPBS (Gibco) for washing. The purified PBMCs were incubated in complete RPMI 1640 medium as mentioned in section **2.4 RPC Isolation and Culture** and incubated at 37°C with 5% CO_2_ (4).

### 2.6 ELISpot assay of induced IFNγ secretion in murine RPCs and porcine PBMCs

We analyzed the secretion of IFNγ *in vitro* following treatment with β-D-glucan with or without inactivated FMDV type O (O PA2) or FMDV type A (A YC) using ELISpot assay kits (R&D Systems, Minneapolis, MN, USA) according to the manufacturer’s protocol. Briefly, isolated murine RPCs or porcine PBMCs (5 × 10^5^ cells/well) were cultured in 96-well polyvinylidene fluoride (PVDF)-backed microplates and treated with 2 μg/mL (final concentration) of inactivated FMDV (O PA2 or A YC) antigen mixed with or without 2.5, 5, 10 μg/mL β-D-glucan for 18 h in an incubator at 37°C with 5% CO_2_. PBS and 2 μg/mL of inactivated FMDV type O (O PA2) or type A (A YC) antigen were used as negative control (NC) and positive control (PC), respectively. Data were obtained using the ImmunoSpot ELISpot reader (Autoimmune Diagnostika GmbH, Strassberg, Germany) (4).

### 2.7 Evaluation of safety in mice vaccinated with the FMD vaccine containing β-D-glucan

We evaluated the safety of the FMD vaccine containing β-D-glucan in mice. The vaccine compositions for the usual vaccination in mice were as follows: purified antigens obtained *via* antigen purification from FMDV type O (O PA2) and type A (A YC) (15 + 15 μg/dose/mL; 1/40 of the dose for pigs), ISA 206 (50% w/w; Seppic, Paris, France), 10% Al(OH)_3_, and 15 μg/mouse Quil-A (InvivoGen, San Diego, CA, USA), with the addition of 100 μg β-D-glucan/dose/mouse in a total volume of 100 μL. Mice were administered with a vaccine equivalent to 5-fold (500 μL) the volume of the usual vaccination dose (100 μL). All mice (n = 5/group) were vaccinated with intraperitoneal (IP) injection into the peritoneum (0 days post-injection [dpi]). To evaluate the safety of the vaccines, survival rates and body weight changes were evaluated up to 7 dpi.

### 2.8 Evaluation of adjuvanticity and early host defense in mice vaccinated with the FMD vaccine containing β-D-glucan

We evaluated the potential of β-D-glucan as a vaccine adjuvant and validated the rapid action of the FMD vaccine containing β-D-glucan in animal experiments. The vaccine compositions for the PC group were as follows: purified antigens obtained *via* Antigen purification from FMDV type O (O PA2) and type A (A YC) (15 + 15 μg/dose/mL; 1/40 of the dose for pigs), ISA 206 (50% w/w; Seppic), 10% Al(OH)_3_, and 15 μg/mouse Quil-A (InvivoGen) in a total volume of 100 μL. Mice in the experimental group received vaccines with the same composition but with the addition of 100 μg β-D-glucan/dose/mouse. Mice in the NC group were administered an equal volume of PBS (pH 7.0). All mice (n = 5/group) were vaccinated with IM injection into the thigh muscle (0 days post-vaccination [dpv]) and challenged with FMDV (100 LD_50_ of O/VET/2013 [ME-SA topotype] or 100 LD_50_ A/Malay/97 [SEA topotype]) by IP injection at 7 dpv. To evaluate the short-term efficacy of the vaccines, survival rates and body weight changes were evaluated up to 7 days post-challenge (dpc) (4, 25).

### 2.9 Assessment of early, mid-, and long-term immune response in mice

We evaluated the efficacy of β-D-glucan as an FMD vaccine adjuvant in establishing early, mid-, and long-term immune responses. Mice (n = 5/group) were vaccinated with the test vaccine *via* the IM route, and blood was collected at 0, 7, 28, 56, and 84 dpv for serological analysis, including structural protein (SP) O and A ELISA and virus-neutralizing (VN) test for O/PKA/44/2008 (O PA2) and A/SKR/YC/2017 (A YC). Unlike NSP ELISA, which measures antibodies and immune responses by live virus infection, SP ELISA measures inactivated antigen-specific antibodies. Serum samples were stored at –80°C until further use (4, 25).

### 2.10 Assessment of early, mid-, and long-term immune responses in pigs

We evaluated the potential of β-D-glucan as an FMDV vaccine adjuvant, and investigated its ability to induce cellular and humoral immune responses and elicit long-term immunity in FMDV type O and type A antibody-seronegative pigs (8–9 weeks-old) according to a previously described method (4). Briefly, pigs were classified into three groups; NC, PC, and Experimental group (n = 5–6/group). The FMD vaccine containing β-D-glucan was composed of purified antigens obtained *via* antigen purification from FMDV type O (O PA2) and type A (A YC) (15 + 15 μg/dose/pig/mL), ISA 206 (50% w/w, Seppic), 10% Al(OH)_3_, 150 μg/dose/pig Quil-A (InvivoGen), and without β-D-glucan (PC group) or with β-D-glucan (experimental group). A single dose was equilibrated to 1 mL. Pigs in the NC group were administered an equal volume of PBS (pH 7.0) (4). After 1^st^ vaccination *via* IM injection, the 2^nd^ vaccination dose was administered at 28 dpv *via* the same route. Blood samples were collected from the vaccinated pigs at 0, 7, 14, 28, 42, 56, and 84 dpv for use in serological assays, including ELISAs (SP O and A), VN test, and immunoglobulin test.

### 2.11 Serological assays

To detect SP antibodies for specific to FMDV serotypes of antigen (O PA and A YC) in sera, we used the VDPro^®^ FMDV type O kit (Median Diagnostics, Gangwon-do, Korea) and PrioCheck™ FMDV type A kit (Prionics AG, Schlieren, Switzerland). The absorbance measurements of the ELISA plate were converted to PI values. When the PI value was ≥40% for the VDPro^®^ FMDV kit or ≥50% for the PrioCheck™ FMDV kit, the animals were considered antibody positive (4). Using FMDV types O (O/PKA/44/2008, O PA2) and A (A/SKR/YC/2017, A YC), virus neutralization tests were performed according to the World Organization for Animal Health (WOAH) manual (16). The collected sera samples were heat-inactivated at 56°C for 30 min, diluted by 2-fold, and incubated with a 100 TCID_50_ (50% tissue culture infective dose) in 50 μL media of FMDV virus (O PA2 or A YC) at 37°C for 1 h. Subsequently, 50 μL of LF-BK cells (10^6^ cells/mL) were added to each well, incubated at 37°C in a 5% CO_2_ atmosphere for 3 d, and the wells were checked for CPE. Antibody titers were evaluated as the log_10_ of the reciprocal antibody dilution required for neutralization of 100 TCID_50_ of viruses in 50% of the wells (25, 27, 28).

When the FMD vaccine containing β-D-glucan was administered, the improved concentration of antigen (O PA2+A YC)-specific antibody (immunoglobulin subtype) titers regardless of the serotype of the antigen in the experimental group compared to the NC and PC groups were investigated. To detect the concentration of antigen (O PA2+A YC)-specific immunoglobulin isotype in sera, ELISAs for porcine IgG, IgA, and IgM (Bethyl Laboratories, Montgomery, TX, USA) were performed according to the manufacturer’s instructions. Briefly, 100 µL of serially diluted standards and serum samples were incubated in each well at approximately 25°C for 1 h, followed by washing four times and incubation with 100 μL of Ig subtype antibodies at approximately 25°C for 1 h. After washing four times, the samples were incubated with 100 μL of HRP solution at approximately 25°C for 30 min. The wells were washed four times and treated with 1× TMB solution (100 μL/well) for 30 min at 25°C to detect peroxidase activity, and the reaction was stopped by adding 2N H_2_PO_4_ (100 μL). Data were obtained at 450 nm using a Hidex 300SL spectrophotometer (Hidex, Turku, Finland) (4, 26).

### 2.12 RNA isolation, cDNA synthesis, and quantitative real-time PCR

We assessed the expression of genes related to the immune response induced by the FMD vaccine containing β-D-glucan as previously described (4, 25). Total RNA was extracted from purified porcine PBMCs (isolated from the whole blood of vaccinated pigs, n = 5–6/group) using TRIzol reagent (Invitrogen, Carlsbad, CA, USA) and RNeasy Mini Kit (QIAGEN, Valencia, CA, USA). RNA was reverse-transcribed to synthesize cDNA using M-MLV RT (Promega, Madison, WI, USA). The cDNA products were amplified on CFX96™ Touch Real-Time PCR Detection System (Bio-Rad Laboratories, Hercules, CA, USA) using iQ SYBR Green Supermix (Bio-Rad Laboratories). Quantitative gene expression levels were normalized to that of HPRT (endogenous housekeeping gene) and presented relative to the control values. The primers used in this study are listed in **Supplementary Table 1**.

### 2.13 Host defense against FMDV type O infection in pigs

We performed a viral challenge experiment to evaluate the efficacy of the FMD vaccine containing β-D-glucan in inducing host defense in pigs. We used FMD type O and A antibody-seronegative animals (8–9 weeks-old), which were classified into three groups (n = 3–4/group). The experimental group was immunized *via* IM injection with a single dose of test vaccine (1 mL) containing β-D-glucan to assess host defense against FMDV infection.

The FMD bivalent test vaccine was composed of FMDV O (O PA2, 15 μg/dose/pig/mL) and FMDV A (A YC, 15 μg/dose/pig/mL) antigen, β-D-glucan (100 μg/dose/pig/mL), ISA 206 (50% w/w; Seppic), 10% Al(OH)_3_, and 150 μg/dose/pig Quil-A (InvivoGen). The PC and NC groups were treated with an equal volume of a commercial FMD vaccine (O Primorsky+A Zabaikalski, ARRIAH-Vac^®^, FGBI ARRIAH, Vladimir, Russia) and PBS, respectively. After vaccination, blood was collected at 0 and 28 dpv to determine antibody titers by performing SP O and A ELISA and VN titers *via* VN test.

Vaccinated pigs were challenged with FMDV type O (O/SKR/JC/2014, Asia topotype; 10^5^ TCID_50_/100 μL) *via* intradermal injection on the heel bulb. Clinical symptoms were monitored daily from 0 to 8 dpc. Sera samples were collected at 0, 2, 4, 6, and 8 dpc by venipuncture (anterior vena cava) and transferred into vacutainer serum tubes (BD Biosciences, San Jose, CA, USA). Oral swab samples were collected daily from 0 to 8 dpc using a BD™ Universal Viral Transport Kit (BD Biosciences). Sera and oral swab samples were stored at –80°C until further analysis.

Clinical scores were determined as follows: 1) reduced appetite (1 point) or no food intake and leftover food from the day before (2 points); 2) lameness (1 point) or reluctance to stand (2 points); 3) presence of heat and pain after palpation of the coronary band (1 point) or not standing on the affected foot (2 points); 4) vesicles on the feet (up to a maximum of 4 points, depending on the number of feet affected); and 5) visible lesions on the tongue (1 point), gums or lips (1 point), or snout (1 point), with a maximum of 3 points (1, 29).

For the viral load assay, viral RNA was extracted from sera and oral swab samples using the QIAcube^®^ HT Pathogen Kit (QIAGEN, Leipzig, Germany), and RT-PCR was performed using a one-step prime-script RT-PCR kit (Bioneer, Daejeon, Korea) according to the manufacturer’s instructions on CFX96 ™ Touch Real-Time PCR Detection System (Bio-Rad Laboratories) for virus quantification (1).

### 2.14 Statistical analysis

All data are expressed as the mean ± standard error of the mean (SEM), unless otherwise stated. Between-group statistical differences were assessed using a two-way ANOVA or one-way ANOVA, followed by Tukey’s *post-hoc* test in either case; all comparisons were parametric. Statistical significance has been denoted as follows: ^*^ *p<*0.05, ^**^ *p<*0.01, ^***^ *p<*0.001, and ^****^ *p*<0.0001. Survival curves were constructed using the Kaplan–Meier method, and differences were analyzed using a log-rank sum test. GraphPad Prism 9.5.0 (GraphPad Software, San Diego, CA, USA) was used for all statistical analyses.

## 3 Results

### 3.1 β-D-glucan with or without inactivated FMDV antigen enhances cellular immune response by inducing IFNγ secretion

**Figure 1:**
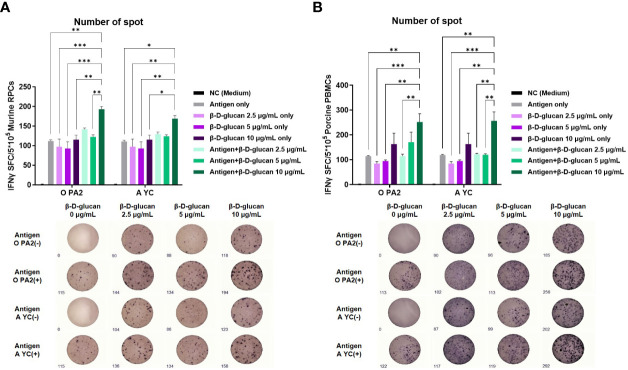
Cellular immune response induced by treatment of β-D-glucan with inactivated foot-and-mouth disease virus (FMDV) type O (O PA2) or A (A YC) antigen in murine resident peritoneal cells (RPCs) and porcine peripheral blood mononuclear cells (PBMCs). IFNγ secretion was assayed to evaluate cellular immune responses induced by test vaccine using the ELISpot assay. Data are presented as mean ± SEM of spot-forming cells (SFCs) from triplicate measurements (n = 3/group). **(A)** IFNγ-secreting cell spots in murine RPCs and **(B)** porcine PBMCs. ^*^ *p* < 0.05, ^**^ *p* < 0.01, ^***^ *p* < 0.001, and ^****^ *p* < 0.0001 (one-way ANOVA followed by Tukey’s test).

To demonstrate the effect of β-D-glucan on cellular immune responses with or without the inactivated FMDV antigen, we evaluated IFNγ secretion *via in vitro* ELISpot assay using murine RPCs isolated from mouse peritoneal lavage fluid and porcine PBMCs isolated from porcine whole blood. β-D-glucan (10 μg/mL) with inactivated FMDV antigen derived from O PA2 or A YC induced significantly higher levels of IFNγ secretion than the negative control (NC, medium only), antigen (O PA2 Ag or A YC Ag), or β-D-glucan alone (2.5, 5, and 10 μg) in murine RPCs and porcine PBMCs (*p <* 0.001, *p* < 0.01, and *p* < 0.05, respectively; **Figures 1A, B**). These results demonstrated that β-D-glucan can induce IFNγ secretion with or without inactivated FMDV antigen and mediate adjuvanticity in FMD vaccines *in vitro*.

### 3.2 FMD vaccine containing β-D-glucan provides potent host defense at the early stage of viral infection in mice

Prior to the efficacy evaluation of the adjuvant, the safety of the FMD test vaccine containing β-D-glucan was evaluated. When mice were intraperitoneally injected with 5 times (500 μL) the usual vaccination volume (100 μL), there was no change in survival rate and weight loss for 7 days, indicating that the FMD vaccine containing β-D-glucan was safe (**Supplementary Figures 3A, B**).

**Figure 2:**
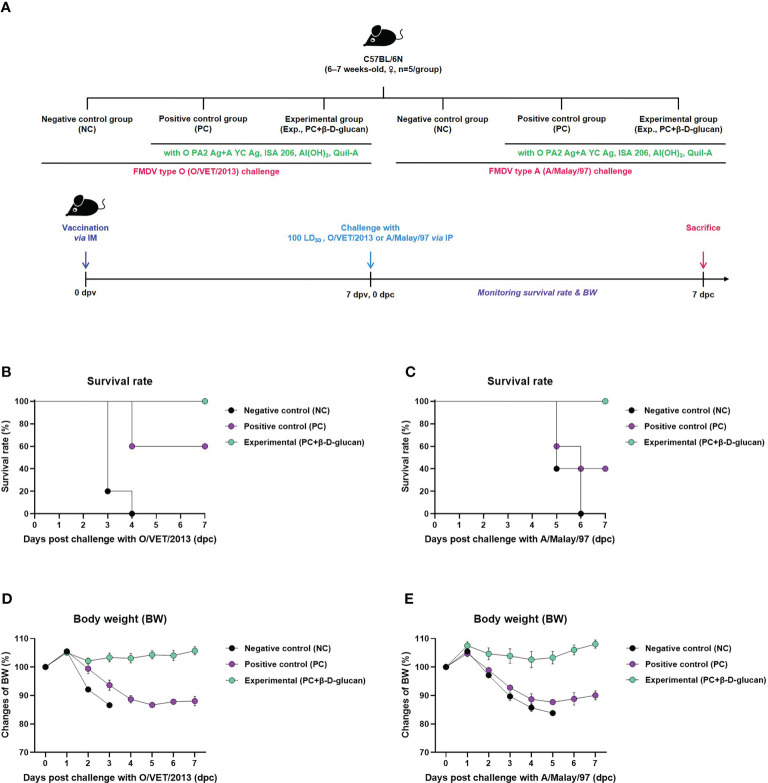
FMD vaccine containing β-D-glucan mediated vaccine efficacy and protective effects in mice. Test vaccines (or PBS) were injected intramuscularly into C57BL/6 mice that were challenged with FMDV O (100 LD_50_ O/VET/2013) or FMDV A (100 LD_50_ A/Malay/97) 7 days post-vaccination (dpv) by intraperitoneal injection. Survival rates and body weights were monitored for 7 days post-challenge (dpc) with the respective viruses. **(A)** Experimental workflow, survival rates post-challenge with **(B)** O/VET/2013 or **(C)** A/Malay/97, and changes in body weight post-challenge with **(D)** O/VET/2013 or **(E)** A/Malay/97. Data are presented as mean ± SEM of triplicate measurements (n = 5/group).

We evaluated the adjuvanticity of β-D-glucan and initial host defense against *in vivo* FMDV infection in mice (**Figure 2A**). We found that mice of the experimental group (receiving the bivalent vaccine containing β-D-glucan and the O PA2+A YC antigens) showed 100% survival against O/VET/2013 (ME-SA topotype) and A/Malay/97 (SEA topotype) (**Figures 2B, C**). No significant body weight change was observed in the experimental group (**Figure 2D, E**). In the PC group that did not receive β-D-glucan, the survival rates against FMDV types O and A were 60% and 40%, respectively, and weight loss increased by more than 10% at 4 dpc with the respective viruses. In the NC group that was treated with PBS, the mortality rate was 100% at 4 and 6 dpc for FMDV type O- and A-infected groups, respectively.

### 3.3 FMD vaccine containing β-D-glucan effectively induces early, mid-, and long-term immune responses in mice and pigs

**Figure 3:**
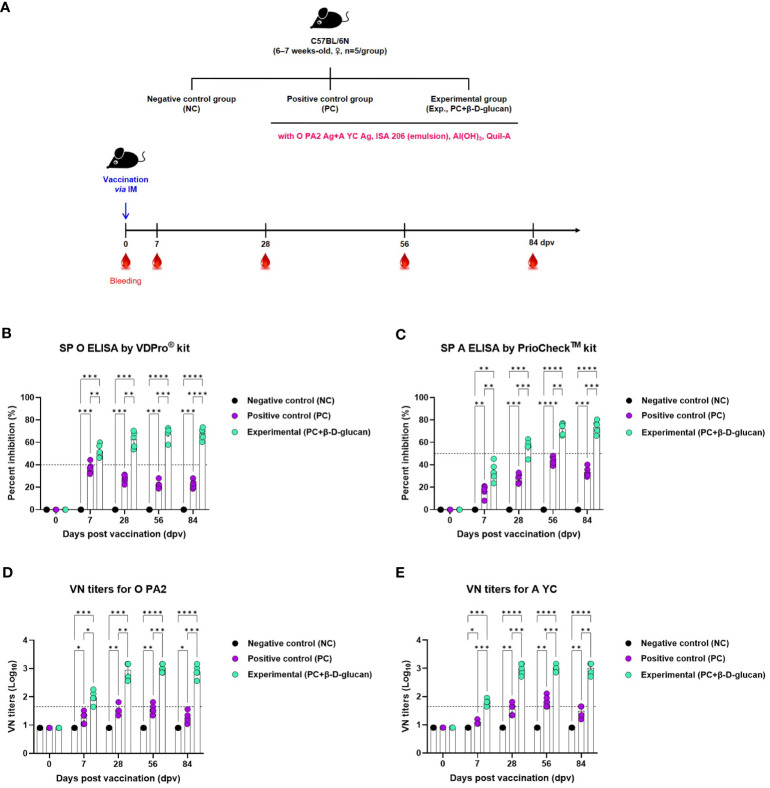
FMD vaccine containing β-D-glucan mediated early, mid-, and long-term immune response in mice. Mice were vaccinated intramuscularly, and blood was collected at 0, 7, 28, 56, and 84 days post-vaccination (dpv) for SP O and A ELISA and virus neutralizing (VN) tests for O/PKA/44/2008 (O PA2) and A/SKR/YC/2017 (A YC). **(A)** Experimental workflow, antibody titers by **(B)** SP O ELSIA for O/PKA/44/2008 (O PA2) or **(C)** SP A ELISA for A/SKR/YC/2017 (A YC), and VN titers for **(D)** O PA2 or **(E)** A YC by VN test. Data are presented as mean ± SEM of triplicate measurements (n = 5–6/group). ^*^ *p* < 0.05, ^**^ *p* < 0.01, ^***^ *p* < 0.001, and ^****^ *p* < 0.0001 (two-way ANOVA with Tukey’s test).

We evaluated the early (7 dpv), mid- (28 and 56 dpv), and long-term (84 dpv) induction of the humoral immune response by the bivalent vaccine (containing O PA2+A YC antigens) with β-D-glucan in mice (**Figure 3A**). We monitored antibody titers over time based on structural protein (SP) O and A ELISA (using VDPro^®^ and PrioCheck™ kits, respectively) and virus-neutralizing (VN) antibody titers by VN test and compared the results between the groups vaccinated with and without β-D-glucan (**Figures 3B–E**, **Supplementary Figures 4A–D**).

Antibody titers were significantly increased in mice of the experimental group compared to that of the PC or NC group at 7, 28, 56, and 84 dpv; the NC group showed no change in antibody titers (**Figures 3B, C**, **Supplementary Figures 4A, B**).

The VN titers for O/PKA/44/2008 (O PA2) and A/SKR/YC/2017 (A YC)—homologous viruses for the O PA2 and A YC antigens—were higher in the experimental group than in the PC or NC group at 7, 28, 56, and 84 dpv; there was no change in VN titers in the NC group (**Figures 3D, E**, **Supplementary Figures 4C, D**).

**Figure 4:**
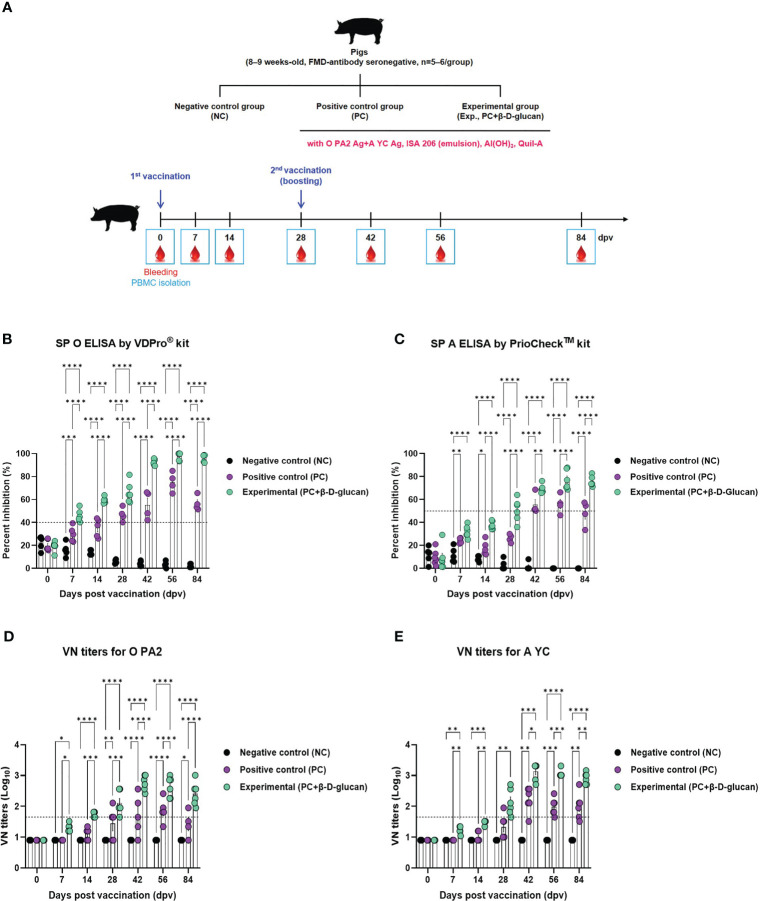
FMD vaccine containing β-D-glucan mediated early, mid-, and long-term immune response in pigs. Single-dose vaccines (1 mL) were administered intramuscularly twice at 28-d intervals (n = 5–6/group). Blood samples were collected at 0, 7, 14, 28, 42, 56, and 84 days post-vaccination (dpv) for serological assays. **(A)** Experimental workflow, antibody titers by **(B)** SP O ELSIA for O/PKA/44/2008 (O PA2) or **(C)** SP A ELISA for A/SKR/YC/2017 (A YC), and VN titers for **(D)** O PA2 or **(E)** A YC by VN test. Data are presented as mean ± SEM of triplicate measurements (n = 5–6/group). ^*^ *p* < 0.05, ^**^ *p* < 0.01, ^***^ *p* < 0.001, and ^****^ *p* < 0.0001 (two-way ANOVA with Tukey’s test).

To verify the induction of the humoral immune response by the bivalent vaccine (containing the O PA2+A YC antigens) with β-D-glucan in pigs, we conducted a target animal experiment using FMD-seronegative pigs (**Figure 4A**) and monitored the induction of early, mid-, and long-term immunity.

The FMDV type O-specific antibody titer was significantly higher in the experimental group than in the PC group at 7 dpv and increased until 28 dpv. After boosting at 28 dpv, antibody titers significantly increased at 42 dpv and remained high until 84 dpv. In contrast, the antibody titer in the PC group increased at a slower rate, compared to that in the experimental group, and the level was only seropositive after 28 dpv. After boosting, the antibody titer reached a maximum at 56 dpv and then decreased rapidly at 84 dpv. No changes were observed in the NC group (**Figure 4B**, **Supplementary Figure 5A**).

When the antibody titer was measured by SP A ELISA, the increase in antibody titer was slower than that measured by SP O ELISA. Nevertheless, 7 dpv after the 1^st^ vaccination, the antibody level in the experimental group was higher than that in the PC group, and the seropositive level was maintained up to 84 dpv. In the PC group, the antibody titer was seronegative up to 28 dpv; only after boosting on 28 dpv did the antibody titer show a seropositive level, although it decreased after 56 dpv and was seronegative at 84 dpv (**Figure 4C**, **Supplementary Figure 5B**).

VN titers for O PA2 and A YC were significantly increased from 7 to 84 dpv in the experimental group compared to the PC group (**Figures 4D, E**, **Supplementary Figures 5C, D**).

**Figure 5:**
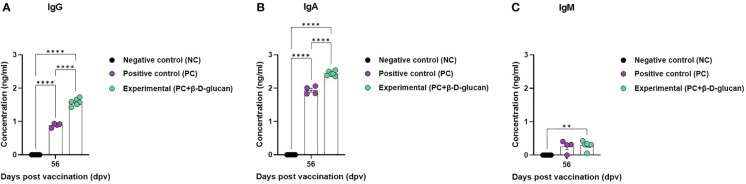
Immune responses mediated by FMD vaccine containing β-D-glucan, based on immunoglobulin subtype (IgG, IgA, and IgM) levels in pigs. Single-dose vaccines (1 mL) were administered intramuscularly twice at 28-d intervals (n = 5–6/group). Blood samples were collected at 0, 7, 14, 28, 42, 56, and 84 days post-vaccination (dpv) for serological assays. **(A)** Antigen [O/PKA/44/2008 (O PA2)+A/SKR/YC/2017 (A YC)]-specific IgG, **(B)** IgA, and **(C)** IgM concentration. Data are presented as the mean ± SEM of triplicate measurements (n = 5–6/group). ^*^ *p* < 0.05, ^**^ *p* < 0.01, ^***^ *p* < 0.001, and ^****^ *p* < 0.0001 (two-way ANOVA with Tukey’s test).

To evaluate the effect of FMD vaccine with or without β-D-glucan as an adjuvant on antigen (O PA2+A YC)-specific IgG, IgA, and IgM levels in pigs, we performed immunoassays using sera from vaccinated pigs at 56 dpv (**Figure 5**). The concentrations of antigen (O PA2+A YC)-specific IgG and IgA were significantly higher in the experimental group than in the PC group. No difference in the concentration of antigen (O PA2+A YC)-specific IgM was observed between the experimental and PC groups. There were no changes in IgG, IgA, or IgM levels in the NC group (**Figures 5A–C**).

### 3.4 FMD vaccine containing β-D-glucan effectively coordinates innate and adaptive immune response by inducing the expression of PRRs, transcription factors, cytokines, and co-stimulatory molecules in porcine PBMCs

**Figure 6:**
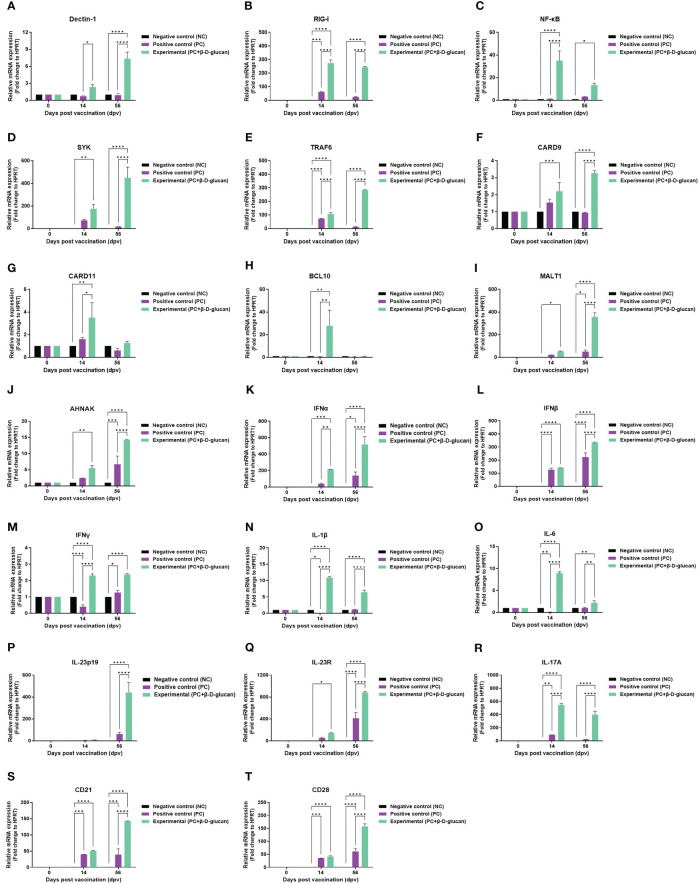
FMD vaccine containing β-D-glucan induced the expression of genes encoding pattern recognition receptors (PRRs), transcription factors, cytokines, and co-stimulatory molecules in porcine PBMCs. Porcine PBMCs were isolated from the whole blood of vaccinated pigs (n = 5–6/group) and analyzed by qRT-PCR. Gene expression levels were normalized to HPRT levels and are presented as ratios relative to control levels. Gene expression levels of **(A)** Dectin-1, **(B)** RIG-I, **(C)** NF-κB, **(D)** SYK, **(E)** TRAF6, **(F)** CARD9, **(G)** CARD11, **(H)** BCL10, **(I)** MALT1, **(J)** AHNAK;, **(K)** IFNα, **(L)** IFNβ, **(M)** IFNγ, **(N)** IL-1β, **(O)** IL-6, **(P)** IL-23p19, **(Q)** IL-23R **(R)**IL-17A, **(S)** CD21, and **(T)** CD28. Data are presented as mean ± SEM of triplicate measurements (n = 5–6/group). ^*^ *p* < 0.05, ^**^ *p* < 0.01, ^***^ *p* < 0.001, and ^****^ *p* < 0.001 (two-way ANOVA followed by Tukey’s test).

To elucidate the mechanism by which β-D-glucan with the FMDV (O PA2+A YC) antigen elicits the immune response, we conducted qRT-PCRs using porcine PBMCs isolated from whole blood (**Figures 6A–T**), as illustrated in **Figure 5A**. We observed changes in the gene expression of PRRs (Dectin-1 and RIG-I), transcription factors [NF-κB p50 subunit (NF-κB), SYK, TRAF6, CARD9, CARD11, BCL10, MALT1, and AHNAK], cytokines (IFNα, IFNβ, IFNγ, IL-1β, IL-6, IL-23p19, IL-23R, and IL-17A), and co-stimulatory molecules (CD21 and CD28) related to the induction and modulation of innate and adaptive immune responses at 0, 14, and 56 dpv. The expression of Dectin-1 and RIG-I was considerably higher in the experimental group compared to that in the PC and NC groups at 14 and 56 dpv (**Figures 6A, B**). The expression of the transcription factors NF-κB (**Figure 6C**), SYK (**Figure 6D**), TRAF6 (**Figure 6E**), CARD9 (**Figure 6F**), MALT1 (**Figure 6I**), and AHNAK (**Figure 6J**) was higher in the experimental group than in the PC and NC groups at 14 and 56 dpv. Notably, the expression levels of SYK (**Figure 6D**), TRAF6 (**Figure 6E**), and MALT1 (**Figure 6I**) varied significantly between the experimental and PC groups at 56 dpv. CARD11 (**Figure 6G**) and BCL10 (**Figure 6H**) expression levels also varied significantly between the experimental and PC groups at 14 dpv.

The expression levels of genes encoding pro-inflammatory cytokines, particularly those of IFNα (**Figure 6K**), IFNβ (**Figure 6L**), and the IL-23p19 (**Figure 6P**)/IL-23R (**Figure 6Q**)/IL-17A (**Figure 6R**) axis, were higher in the experimental group than in the PC and NC groups at 14 and 56 dpv, indicating a strong induction of host defense. The expression level of IFNγ (**Figure 6M**) was somewhat lower than that of IFNα and IFNβ but significantly higher in the experimental group than in the PC and NC groups at 14 and 56 dpv. IL-1β (**Figure 6N**) and IL-6 (**Figure 6O**) expression levels in the experimental group were also markedly higher than those in the PC and NC groups at 14 and 56 dpv.

The expression levels of the co-stimulatory molecules CD21 (**Figure 6S**) and CD28 (**Figure 6T**) were significantly higher in the experimental group than in the PC and NC groups.

### 3.5 Single-shot of FMD vaccine including β-D-glucan drives potent host defense against FMDV infection in pigs

**Figure 7:**
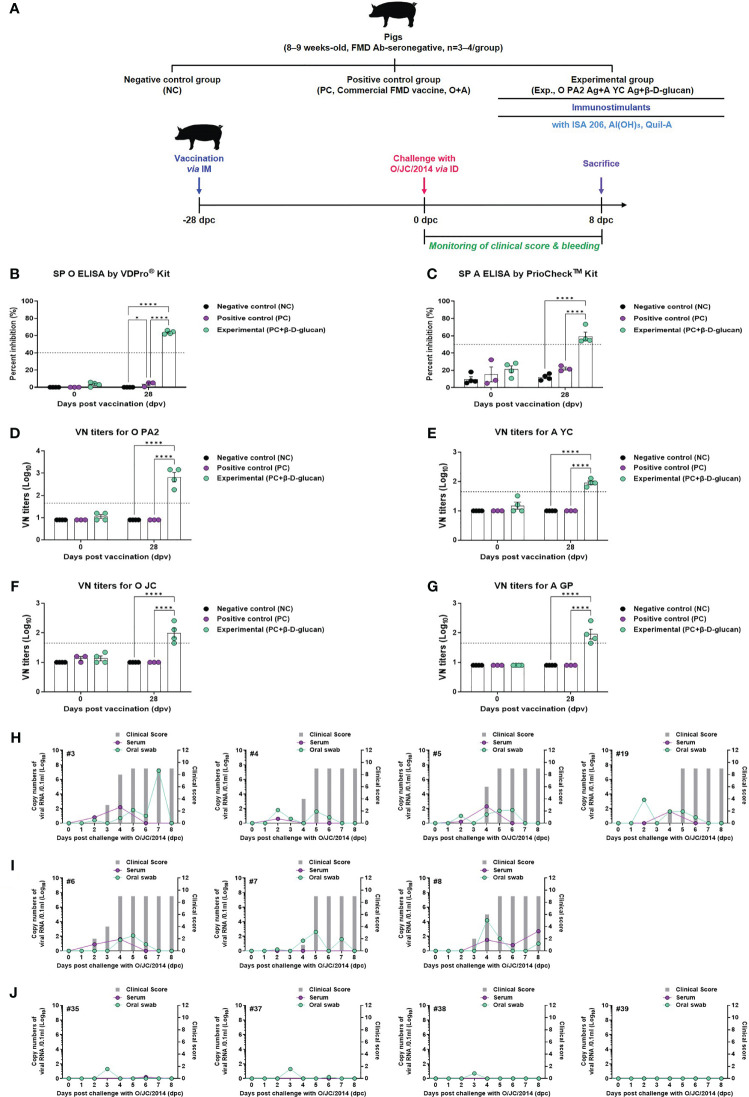
FMD vaccine containing β-D-glucan mediated host defense against FMDV O infection in pigs. Blood samples were collected at 0 and 28 days post-vaccination (dpv) for serological assays. Vaccinated pigs (n = 3–4/group) were challenged with FMDV type O (O/SKR/JC/2014) at 28 dpv. **(A)** Experimental workflow. Antibody titers by **(B)** SP O ELSIA or **(C)** SP A ELISA. VN titers for **(D)** O PA2 or **(E)** A YC or **(F)** O JC or **(G)** A GP by VN test. Clinical score and viral load in serum and oral swabs **(H)** from NC pigs (n = 4/group), **(I)** PC pigs (commercial FMD vaccine, n = 3/group), and **(J)** experimental group pigs (O PA2 + A YC + β-D-glucan, n = 4/group) against FMDV type O (O/SKR/JC/2014). The left Y-axis of the graph shows the amount of virus in sera and oral swab samples as log_10_ values; the right Y-axis shows the clinical index as the maximum value of 10 points. Data are presented as mean ± SEM of triplicate measurements (n = 3–4/group).

To validate the β-D-glucan-induced innate and adaptive immune responses in pigs, FMD antibody-seronegative animals were immunized with a single dose of the bivalent (O PA2 + A YC antigen [O+A]) test vaccine with β-D-glucan and challenged with FMDV type O (O/SKR/JC/2014, Asia topotype) at 28 dpv (1). The PC and NC groups were treated with an equal volume of commercial FMD vaccine (ARRIAH-Vac^®^) and PBS, respectively, and challenged with FMDV type O, following the same schedule (**Figure 7A**).

Antibody titers by SP O and A ELISA and VN titers for FMDV types O and A were measured at 28 dpv (**Figures 7B–G**, **Supplementary Figure 6A**). The antibody titers measured by both SP O and A ELISA were significantly higher in the experimental group than in the PC and NC groups (**Figures 7B, C**).

VN titers for the virus homologous for O/PKA/44/2008 (O PA2) and A/SKR/YC/2017 (A YC) showed similar results to those of the antibody titers measured by SP O ELISA and SP A ELISA at 28 dpv (**Figures 7D, E**). The VN titer for the virus heterologous for O/SKR/JC/2014 (O JC) and A/SKR/GP/2018 (A GP) was significantly higher in the experimental group than in the PC and NC groups at 28 dpv (**Figures 7F, G**). We analyzed several diseases parameters following challenge with FMDV type O at 28 dpv (0 dpc), including clinical symptoms, viremia in sera, and viral titers in oral swabs, until 8 dpc. The NC group (animals #3, #4, #5, and #19) showed all (4/4) of the typical clinical symptoms of FMD, and high levels of viral titers were detected in sera and oral swabs (**Figure 7H**). In the PC group, all animals (3/3) showed the clinical symptoms of FMDV type O infection (animal #6, #7, and #8), indicating no protection. Viremia from sera and viral titers in oral swabs were detected between 1 and 4 (log_10_) for FMDV type O infection (**Figure 7I**). In contrast, the animals in the O PA2 + A YC + β-D-glucan group (animals #35, #37, #38, and #39) did not show any clinical symptoms of FMD, and there was no viral release from the sera and oral cavities (**Figures 7J**), indicating potent protection against infection.

## 4 Discussion

The FMDV, a single-stranded RNA(+) virus, is highly contagious and incredibly well adapted for rapid long-distance transmission. Therefore, infected livestock are slaughtered and buried to prevent transmission, which causes considerable damage to the livestock industry. For these reasons, the WOAH has designated FMD as the most dangerous class A virus among livestock infectious diseases.

Most countries at risk of FMD outbreaks have implemented vaccinations to prevent and control disease spread. Current FMD vaccines use a combination of inactivated viral antigens and oil adjuvants (emulsions) to enhance their efficacy. However, commercial FMD vaccines have several limitations, including the delayed induction of antibody secretion and short-lived persistence of antibody titers. In addition, commercial FMD vaccines show lower antibody titers in pigs than in cattle, and despite high antibody levels, protection against viral infection is imperfect across species. Above all, the vaccines are prone to cause side effects during injection (30, 31).

Adverse reactions caused by the oil adjuvant at the site of vaccination have caused enormous economic losses in the Korean pig industry (32). Vaccine safety remains a major challenge in the development of novel adjuvants. Aluminum gels and oil emulsions remain the gold standards for adjuvants. Based on the findings in our previous study (2), we assessed the performance of β-D-glucan as a novel FMD vaccine adjuvant to modulate innate and adaptive immune responses and simultaneously enhance cellular and humoral immune responses in target animals, specifically pigs.

To evaluate the cellular immune response elicited by the β-D-glucan with or without O PA2 and A YC antigens, we measured the levels of IFNγ-secreting cells using ELISpot assay in murine RPCs and porcine PBMCs. RPCs include innate immune cells, such as APCs, including DCs, MΦs, monocytes, and unconventional T cells (gamma delta [γδ] T cells, mucosal-associated invariant T [MAIT] cells, and invariant natural killer [iNKT] T cells). Therefore, RPCs are especially suitable for studying cellular and systemic immune responses (1). PBMCs comprise lymphocytes (T, B, and NK cells), monocytes, and DCs. IFNγ is primarily secreted by T helper (especially Th1), cytotoxic T, NK, and MΦ cells. IFNγ is a major parameter in evaluating initial host defenses, as it is a crucial autocrine signal for APCs in the innate immune response and a paracrine signal in the adaptive immune response (33). Based on the ELISpot results, the levels of IFNγ-secreting cells (number of spots) increased significantly in the O PA2+β-D-glucan or A YC+β-D-glucan-treated group compared to that in the PC groups for both FMD antigen types.

The level of IFNγ-secreting cells in both murine RPCs and porcine PBMCs increased in a dose-dependent manner, with no cytotoxicity observed (**Figure 1**). These results suggest that β-D-glucan is safe even when administered at high doses. We conducted additional experiments with double the dose of β-D-glucan (20 μg/mL) to determine the optimal dosage and found that the level of IFNγ remained consistent between doses (data not shown); thus, the optimal dose *in vitro* was confirmed to be 10 μg/mL.

We evaluated the safety of FMD vaccine containing β-D-glucan as an adjuvant in mice (**Supplementary Figure 3**). In addition, when the FMD test vaccine containing β-D-glucan was intramuscularly injected into pigs, the target animal, vaccine side effects, such as animal death and weight gain, were not observed in the experimental group compared to the NC and PC groups. Therefore, the FMD vaccine containing β-D-glucan was considered safe.

We evaluated the efficacy of β-D-glucan in mice to confirm the early protective effects against FMDV type O and A and found that the FMD vaccine containing β-D-glucan showed potent host defense against both variants, reflecting its potential as an effective adjuvant that elicits a stronger initial immune response than conventional FMD vaccines (**Figure 2**).

To confirm the induction of the humoral immune response, mice and pigs were vaccinated and their antibody and VN titers were assessed during the early, mid-, and long-term immune response. Both antibody and VN titers for O PA2 and A YC were higher in the experimental group compared with those in the PC group, and the levels were also maintained for an extended period in both mice and pigs (**Figures 3**, **4**, **Supplementary Figure 4, 5**).

With the VDPro^®^ and PrioChek™ kit, antibody titers are considered seropositive when percent inhibition is ≥40% or ≥50%, respectively. FMD vaccines have been reported to achieve VN titers >1.65 (log_10_) (34), which is associated with effective host defense according to the Korean FMD vaccine evaluation criteria. In this study, the experimental group maintained a VN titer >2 log_10_ up to 84 dpv for both FMDV types O and A, whereas the PC group only maintained a seropositive VN titer up to 56 dpv. These findings suggest that β-D-glucan may play a pivotal role in eliciting an early immune response that is maintained in the long-term and provides cross-protection against FMDV type O and A infections.

Next, we evaluated the induction of antigen (O PA2+A YC)-specific IgG, IgA, and IgM, which are markers of general neutralizing antibodies, mucosal immunity, and natural (innate) immunity, respectively, in addition to the specific response to β-D-glucan-mediated FMDV structural proteins (35). Based on the immunoglobulin isotype ELISA, the experimental group had a significantly higher IgG concentration than the PC group (**Figure 5**). Vidarsson et al. reported that IgG antibodies play a pivotal role in the secondary immune response, as these antibodies are produced following class switching and maturation of the antibody response (36). Therefore, our results demonstrated that β-D-glucan induces a memory immune response by effectively eliciting a humoral immune response.

Miyamoto et al. designed an active targeted delivery system using β-D-glucan, which interacts with immune cells, such as DCs, MΦs, neutrophils, and T cells, and is not digested *in vivo* (37). This suggests that the novel FMD vaccine containing β-D-glucan can be administered not only *via* the IM route but also *via* mucosal (such as nasal and oral) routes, which would minimize the risk of side effects.

We determined the gene expression stimulated by FMD vaccine containing β-D-glucan in pigs by qRT-PCR. One of the most significantly expressed genes, Dectin-1, induces CARD9 expression through SYK after recognizing β-D-glucan. CARD9 is expressed in myeloid cells, MΦs, and DCs, and CARD9 complexes with BCL10 and MALT1 contribute to innate and adaptive immune responses to viral infections (38, 39). The CBM complex also upregulates the expression of NF-κB in DCs, leading to the secretion of cytokines, such as IL-1β, IL-6, and IL-23p19 (40, 41). IL-23 binds to IL-23R on the surface of unconventional T cells, thereby promoting the differentiation of Th17 cells to induce the secretion of IL-17A (42). The experimental group containing β-D-glucan induced a significant increase in IL-17A expression compared to that in the PC group (43).

IL-17A is an important indicator of the degree of stimulation and activation of unconventional T cells. Conventional and unconventional T cells such as CD4^+^, CD8^+^, Th17, γδ T, iNKT, MAIT, and innate lymphoid cells (ILCs) can induce the production of IL-17A (44). Non-T cells, such as neutrophils, can also induce IL-17A production under certain conditions (45). IL-17A can promote the production of the cytokine IFNγ and stimulate IL-6 induction through NF-κB expression in fibroblasts (46, 47). IL-17A contributes to neutrophil recruitment and promotes the formation of neutrophil extracellular traps (NETs) (48). Thus, the IL-23/IL-17A axis plays a critical role in the initial host defense against pathogen (viral and bacterial) infection by stimulating Th17 cells and unconventional T cells (49). CARD9 also stimulates Th1 cells to promote IFNγ expression and plays a pivotal role in enhancing innate and adaptive immunity (50–52). CARD9 enhances signaling from innate immune cells to T cells, further promoting neutrophil and MΦ recruitment to sites of viral infection. Thus, CARD9-mediated signaling contributes to a variety of cellular immune functions in innate and adaptive immunity.

qRT-PCR revealed that the experimental group exhibited significant differences in the expression of transcription factors, proinflammatory cytokines, and co-stimulatory molecules, compared to the PC group (**Figure 6**) (4, 19). In contrast, although β-D-glucan is a Dectin-1 ligand, the FMD vaccine induces RIG-I expression, promoting the expression of type I IFNs that participate in initial host defense (53). IFNα and IFNβ are secreted by many cell types, including lymphocytes (NK, B, and T cells), MΦs, and DCs, and stimulate MΦs and NK cells to elicit an antiviral response (54). Therefore, β-D-glucan induced various proinflammatory cytokines and activated unconventional T cells by inducing the formation of the CARD9-BCL10-MALT1 complex through CARD9 expression.

AHNAK binds and interacts with the cytoplasmic β subunit (Cavβ), which regulates the expression level and gating kinetics of L-type voltage-gated calcium channels (LVGCCs) (55, 56). Cavβ belongs to the membrane-associated guanylate kinase (MAGUK) protein group (57, 58). MAGUK is also part of the CARD family (CARD10, CARD11, and CARD14) and is involved in the assembly of CBM complexes (59–61). This suggests that AHNAK may interact with Cavβ and consequently be involved in CARD family-mediated signaling.

Our experimental results confirmed the effects of β-D-glucan in eliciting the immune response by challenge experiments after 28 dpv in pigs; the experimental group showed potent host defense, with no clinical symptoms, viremia, or viral titers detected, unlike the PC and NC groups (**Figure 7**, **Supplementary Figure 6**). These results suggest that novel FMD vaccines containing β-D-glucan drive robust cellular and humoral immune responses by modulating the host’s innate and adaptive immunity in the early stage of viral infection. It is also possible that the vaccine suppresses viral proliferation and induces antiviral effects by promoting the expression of cytokines, such as type I IFN.

Collectively, our findings suggest that the novel adjuvant β-D-glucan induces a potent immune response by stimulating innate immune cells, such as DCs, MΦs, neutrophils, NK cells and unconventional T cells, and adaptive immune cells such as T cells and B cells, to coordinate innate and adaptive immune responses. β-D-glucan also contributes to cellular and humoral immune responses by upregulating the expression of proinflammatory cytokines, co-stimulatory molecules, and transcription factors, thereby eliciting host defenses and long-term immunity. Although this study demonstrates that β-D-glucan is effective against FMD, we hypothesize that β-D-glucan is a promising adjuvant in the development of ideal vaccines against porcine reproductive and respiratory syndrome virus (PRRSV), African swine fever virus (ASFV), and severe acute respiratory syndrome coronavirus 2 (SARS-CoV-2) through the orchestration of innate and adaptive immune response of the host. In future studies, we intend to evaluate the potential for application *via* mucosal routes, which could contribute to the development of bait vaccines for the prevention and treatment of diseases of international concern.

## Data availability statement

The original contributions presented in the study are included in the article/**Supplementary Material**. Further inquiries can be directed to the corresponding author.

## Ethics statement

The animal study was conducted in accordance with institutional guidelines and was approved by the Animal and Plant Quarantine Agency (APQA) Ethics Committee (accreditation number: IACUC-2022-659).

## Author contributions

Conceptualization, MJL; methodology, MJL; software, HWK and MJL; validation, HWK and MJL; formal analysis, HWK and MJL; investigation, HWK, M-KK, SHP, SYH, DHK, SYP, and MJL; resources, S-MK, J-HP, and MJL; writing—original draft preparation, HWK and MJL; writing—review and editing, HWK, Y-JK, S-MK, and MJL; visualization, HWK and MJL; supervision, MJL; project administration, MJL; funding acquisition, MJL. All authors contributed to the article and approved the submitted version.
