# Supplementary material for: Dectin-1 signaling coordinates innate and adaptive immunity for potent host defense against viral infection
Source: Front Immunol. 2023 Jun 2;14:1194502. doi: 10.3389/fimmu.2023.1194502 (PMC10272586; doi:10.3389/fimmu.2023.1194502)
Supplement: Supplementary file 1 [file DataSheet_1.pdf]

Supplementary Material

Dectin-1 signaling coordinates innate and adaptive immunity for potent host defense against viral infection

Hyeong Won Kim, Mi-Kyeong Ko, So Hui Park, Seong Yun Hwang, Dong Hyeon Kim, Sun Young Park, Young-Joon Ko, Su-Mi Kim, Jong-Hyeon Park, Min Ja Lee*

* **Correspondence:** Min Ja Lee: herb12@korea.kr

1 Supplementary Figures and Tables

1.1 Supplementary Figures

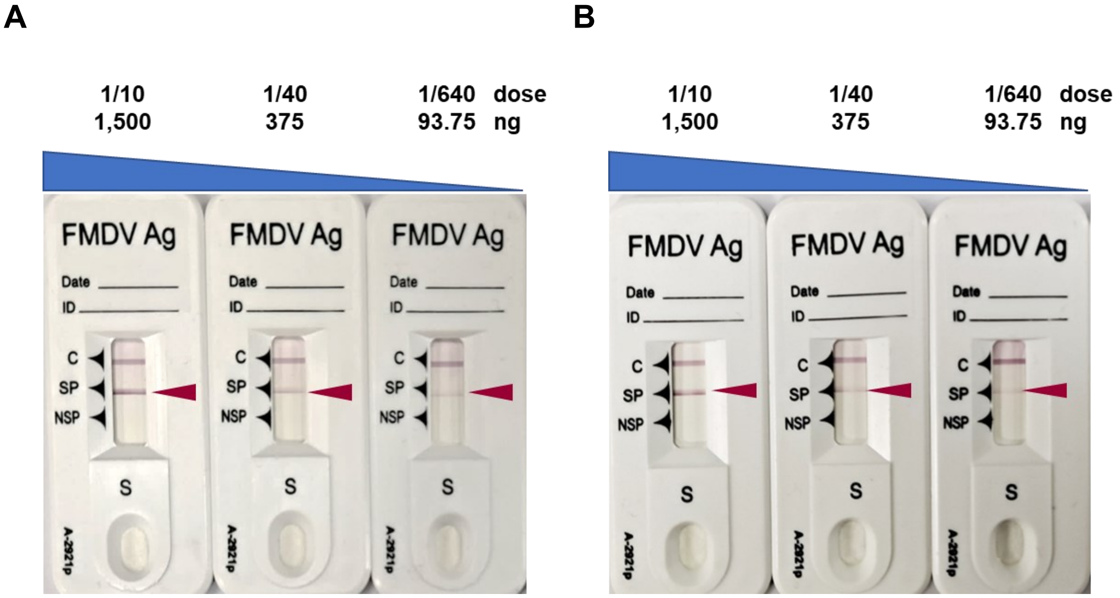

Supplementary Figure 1. Detection of the inactivated FMDV (O PA2 and A YC) antigens in serial dilutions using a rapid test kit for type O or type A. Structural proteins (SPs) of the purified antigen, expressed by cells infected with the FMDV O PA2 and A YC antigens, and confirmed by rapid antigen kits (PBM kits). The images show band formation for the SPs and no band formation for the non-structural proteins (NSPs) of FMDV: (**A**) O PA2, (**B**) A YC.

A B

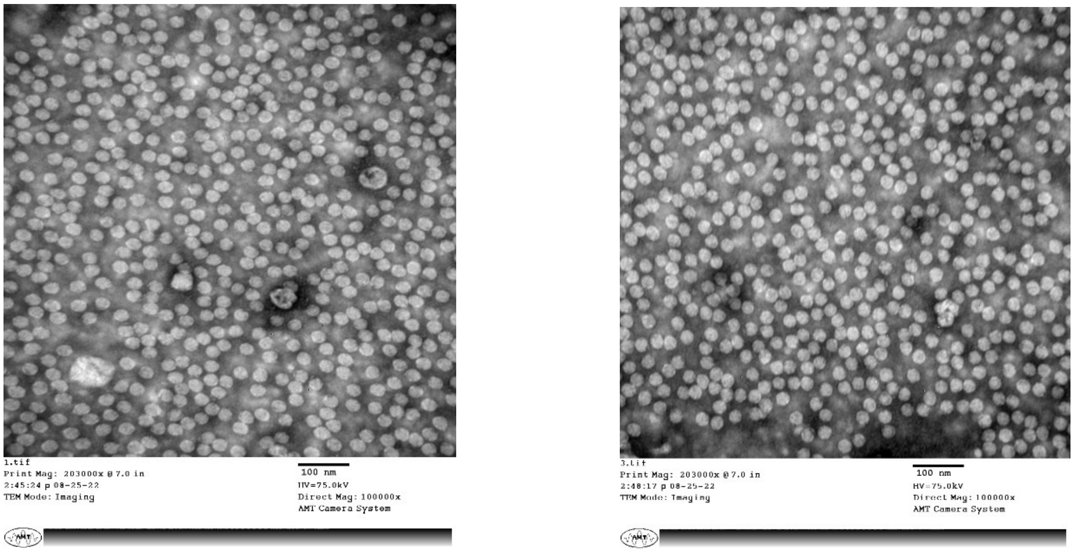

Supplementary Figure 2. Electron microscopic examination of the inactivated antigen of the FMD vaccine strain. The virus particle (146S) as characterized by transmission electron microscope (TEM) imaging. (**A**) O PA2, (**B**) A YC.

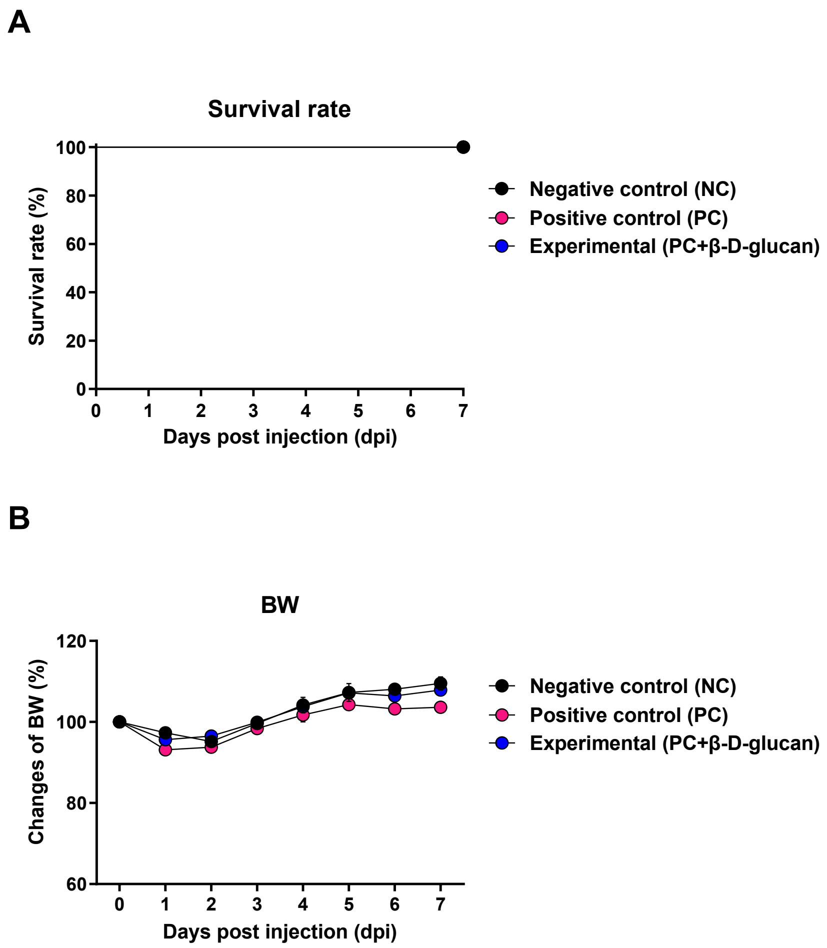

Supplementary Figure 3. Safety of FMD vaccine containing β-D-glucan as an adjuvant in mice. To test the safety of the FMD vaccine containing β-D-glucan, mice were administered a vaccine equivalent to 5 times (500 μL) the dose (100 μL) of 1/10 of the target animal (cow or pig) via intraperitoneal (IP) injection into the mouse peritoneal cavity, and the survival rate and change of body weight was monitored up to for 7 days post-injection (dpi). (**A**) Survival rate, (**B**) Change of body weight.

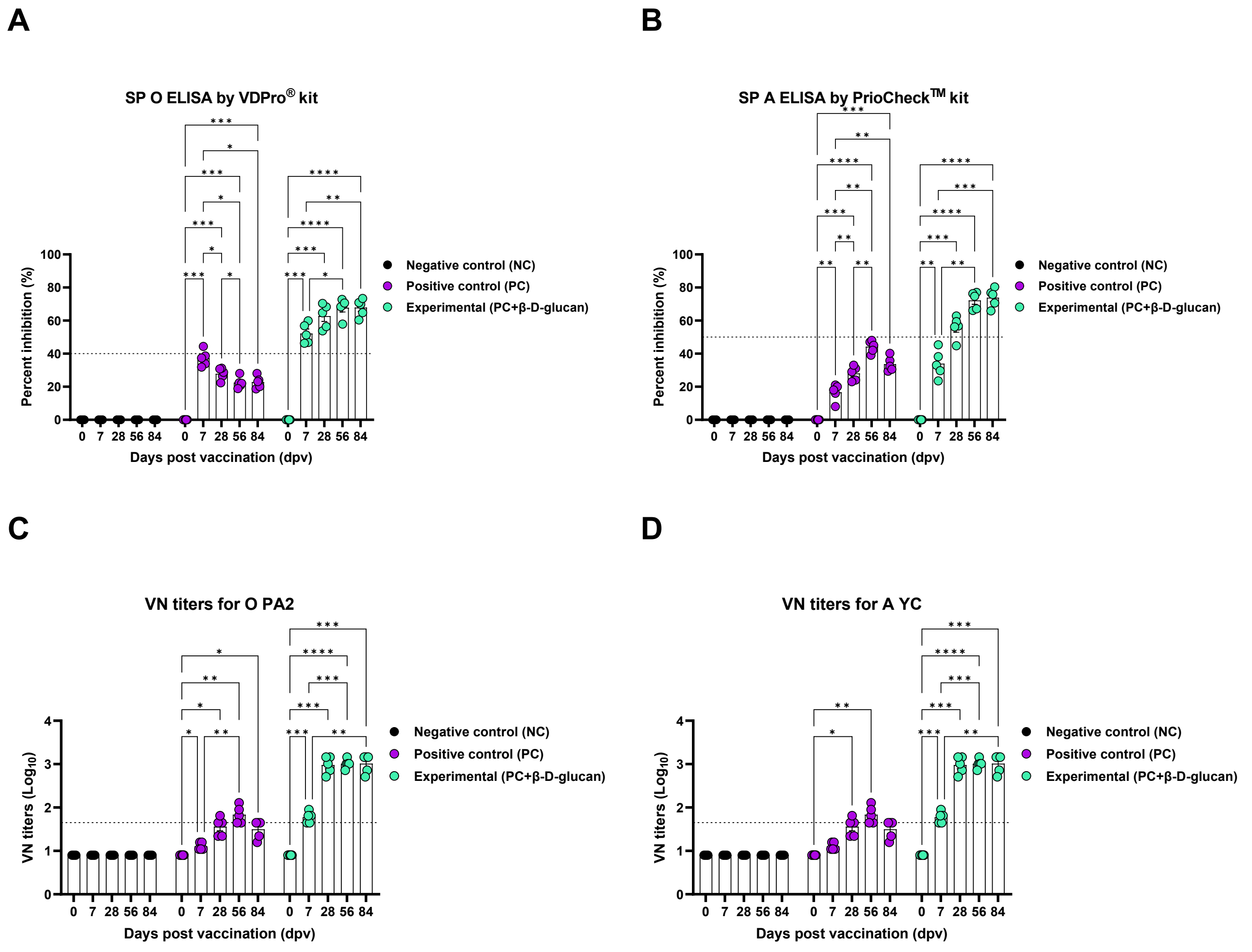

Supplementary Figure 4. Statistical analysis of time-dependent antibody titers and VN titers for each group in Figure 3.

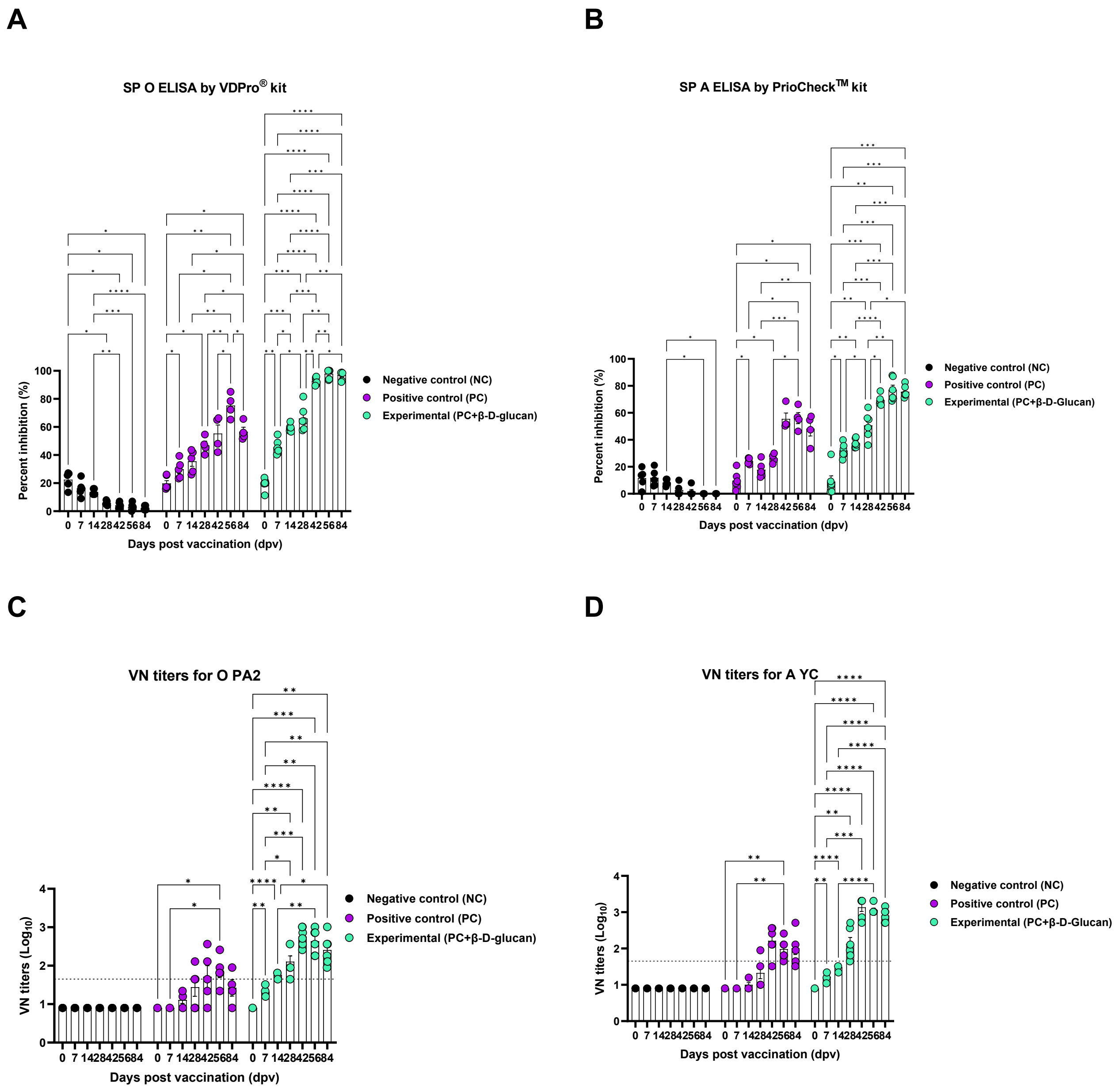

Supplementary Figure 5. Statistical analysis of time-dependent antibody titers and VN titers for each group in Figure 4.

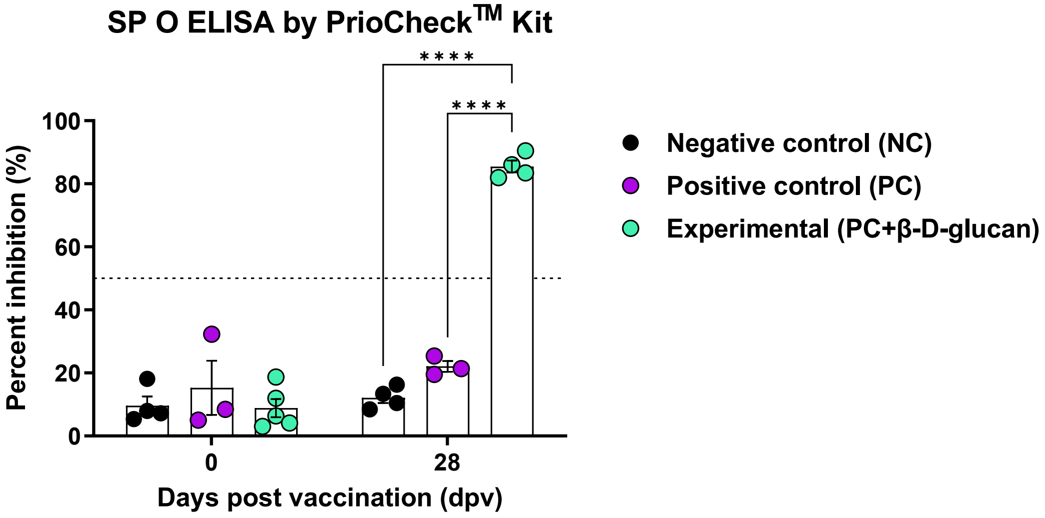

Supplementary Figure 6. β-D-glucan with FMD vaccine-mediated antibody titers by SP O ELISA using PrioCheck™ kit in pigs. For the challenge experiments, FMDV type O and type A antibody-seronegative pigs (8–9 weeks-old, n = 5–6/group) were administered FMD vaccine including FMDV type O (O PA2) and type A (A YC) antigen (15+15 μg/dose/mL, one dose for cattle and pig use) with ISA 206 (oil-based emulsion, 50%, w/w), 10% $Al(OH)_3$, and 150 μg Quil-A. One milliliter vaccine was prepared as a single dose and introduced into the animals via intramuscular (IM) injection. The positive control (PC) and negative control (NC) groups of pigs were treated with an equal volume of commercial FMD vaccine (O Primorsky+A Zabaikalski, ARRIAH-VAC®, FGBI ARRIAH, Vladimir, Russia) and PBS, respectively, via the same route. Blood samples were collected at 0 and 28 d post-vaccination (dpv) in pigs for serological assays. Vaccinated pigs were challenged with FMDV type O (O/SKR/JC/2014) on the heel bulb at 10^5 $TCID_{50}$/100 μL at 28 dpv. The plot represents Ab titers by SP O ELSIA using PrioCheck™ kit. The data represent the mean ± SEM of triplicate measurements (n=5–6/group). $^{****}p < 0.0001$ (two-way ANOVA with Tukey's test).

1.2 Supplementary Table

Supplementary Table 1. List of primer sequences for qRT-PCR.

Target	Forward/Reverse	Sequence (5'- 3')	Length (mer)
Dectin-1	Dectin-1 F	ACAGCTCCAAAGAGCTGGAA	20
	Dectin-1 R	CCAGCTCTTTGGAGCTGTCTA	20
RIG-I	RIG-I F	GCACCTCATACTTACAGCCCA	21
	RIG-I R	CCACAACCAGTAGGAGCACAT	21
NF-κB	NF-κB F	TCGCTGCCAAAGAAGGACAT	20
	NF-κB R	AGCGTTCAGACCTTCACCGT	20
SYK	SYK F	CCAACCACTTGCCCTTCTTC	20
	SYK R	ATGGTGTAGTGATGCGCCTT	20
TRAF6	TRAF6 F	TCGCAGTAGCTCCTGTACCT	20
	TRAF6 R	AGCTCCCGGATTTGATGGTC	20
CARD9	CARD9 F	CCGCAGCTCTACAAGAAGGT	20
	CARD9 R	TCTGCAGCTTCATCACCTCG	20
hCARD11	CARD11 F	TGAACGAGGTCATCAAGCTG	20
	CARD11 R	AGCGTCAGCTGCTTCTTCTC	20
BCL10	BCL10 F	ATGGAGCCCGCCGCGCCGTC	20
	BCL10 R	GCTATGATTTTTTCACACAG	20
MALT1	MALT1 F	GTTGGAAGCCCCATTCCACA	20
	MALT1 R	ACTCCACTGCCTCATCTGTTC	21
AHNAK	AHNAK F	CACCATCACCGTGACTCGAA	20
	AHNAK R	AGTTCGTGCCGTGGAATCTT	20
IFNα	IFNα F	CATCTGCTCTCTGGGCTGTG	20
	IFNα R	TGAGGGGATCCAAAGTCCCT	20
IFNβ	IFNβ F	TGCAACCACCACAATTCCAGA	21
	IFNβ R	GGTTTCATTCCAGCCAGTGC	20
IFNγ	IFNγ F	GCCATTCAAAGGAGCATGGAT	21
	IFNγ R	CTGATGGCTTTGCGCTGGAT	20
IL-1β	IL-1β F	AGCCAGTCTTCATTGTTCAGGT	22
	IL-1β R	TCATCTCTTTGGGGCCATCAG	21
IL-6	IL-6 F	CTGCAGTCACAGAACGAGTG	20
	IL-6 R	CGGCATCAATCTCAGGTGCC	20

Supplementary Table 1 (continued)

Target	Forward/Reverse	Sequence (5'- 3')	Length (mer)
IL-23p19	IL-23p19 F	CCATATCCAGTGCGGGGATG	20
	IL-23p19 R	AGGCCTTGGTGGATCCTTTG	20
IL-23R	IL-23R F	TCCCTCATTGCAAAGCACAA	20
	IL-23R R	GCATCTCCTCTTGCAAGCAAAT	22
IL-17A	IL-17A F	CTCGTGAAGGCGGGAATCAT	20
	IL-17A R	GGTGTGCTCCGGTTCAAGAT	20
CD21	CD21 F	TGCCATGCCTACAAAGCTGA	20
	CD21 R	GTAGTAACCAGGGCGGCATT	20
CD28	CD28 F	TCAAAGGAGTTCCGGGCATC	20
	CD28 R	CTGAAGCAGGCGGGAGTAAT	20
HPRT	HPRT F	CCCAGCGTCGTGATTAGTGA	20
	HPRT R	GCCGTTCAGTCCTGTCCATA	20
